# Primary Uterine Inertia (PUI) in Dogs Is Associated with Impaired Placental Availability of Factors Involved in the Parturition Cascade

**DOI:** 10.3390/ani15203043

**Published:** 2025-10-20

**Authors:** Marianne Steiner, Gerhard Schuler, Bianca L. Frehner, Iris M. Reichler, Sandra Goericke-Pesch, Orsolya Balogh, Miguel Tavares Pereira, Mariusz P. Kowalewski

**Affiliations:** 1 Institute of Veterinary Anatomy, Vetsuisse Faculty, University of Zurich, CH-8057 Zurich, Switzerland; marianne.steiner2@uzh.ch (M.S.); m.a.tavarespereira@uu.nl (M.T.P.); 2 Clinic for Obstetrics, Gynecology and Andrology of Large and Small Animals, Justus-Liebig-University, 35392 Giessen, Germany; gerhard.schuler@vetmed.uni-giessen.de; 3 Clinic for Reproductive Medicine, Vetsuisse Faculty, University of Zurich, CH-8057 Zurich, Switzerlandiris.reichler@uzh.ch (I.M.R.); 4 Unit for Reproductive Medicine, Clinic for Small Animals, University of Veterinary Medicine Hannover, 30559 Hannover, Germany; sandra.goericke-pesch@tiho-hannover.de; 5 Clinical Sciences, Virginia-Maryland College of Veterinary Medicine, Blacksburg, VA 24061, USA; obalogh@vt.edu; 6 Anatomy and Physiology, Department Clinical Sciences, Faculty of Veterinary Medicine, Utrecht University, 3584 CL Utrecht, The Netherlands

**Keywords:** dog (*Canis lupus familiaris*), parturition cascade, primary uterine inertia (PUI), placenta, progesterone signaling

## Abstract

**Simple Summary:**

In dogs, the parturition is initiated by decreased progesterone (P4) signaling in the placenta, mediated through its receptor, PGR, located in the maternal part of the placenta (decidual cells). This leads to increased production of PGF2α in fetal placental cells (trophoblasts), inducing luteolysis and placentolysis (i.e., the breakdown of the corpus luteum and placental function), and triggering uterine contractions. Glucocorticoids (e.g., cortisol) appear to contribute to the initiation of parturition by affecting P4-PGR signaling through the glucocorticoid receptor (*GR*/*NR3C1*). The underlying causes of primary uterine inertia (PUI), a common cause of dystocia (difficult labor) in dogs, are unclear. This study compared placentae from dogs with PUI to those undergoing normal prepartum luteolysis (LUT) to investigate whether abnormal placental signaling could contribute to PUI. PUI placentae had no major changes in the prostaglandin-related enzymes (PTGS2, PTGES, HPGD), but had reduced expression of PGF2α synthase (PGFS/AKR1C3) and increased prostaglandin transporter (PGT), indicating impaired PGF2α activity. PGR was increased, although the number of maternal decidual cells remained unchanged. *GR*/*NR3C1* levels were lower in PUI. In conclusion, PUI may be associated with disrupted hormonal signaling in the placenta involving the P4-PGR and glucocorticoid pathways, resulting in impaired parturition in dogs.

**Abstract:**

The canine parturition cascade involves decreased placental progesterone (P4) signaling mediated through its nuclear receptor PGR in decidual cells, leading to increased trophoblast production of PGF2α that promotes luteolysis, placentolysis, and myometrial contractility. A local role for glucocorticoids in initiating parturition through increased placental availability of cortisol and glucocorticoid receptor (*GR*/*NR3C1*), possibly affecting P4-PGR signaling, has been suggested. Primary uterine inertia (PUI) is a major cause of canine dystocia, but its pathophysiology remains unclear. Here, we hypothesized that dysregulated placental signaling could contribute to PUI. The availability of parturition cascade-related factors was assessed in placentae of dogs with PUI and during physiological prepartum luteolysis (LUT). Compared with LUT, PUI had no significant changes in prostaglandin-related factors *PTGS2*, *PTGES*, and *HPGD* (*p* > 0.05), but had lower PGF2α synthase *PGFS*/*AKR1C3* (*p* < 0.001), and higher *PGT* abundance (*p* < 0.001). PUI had increased PGR transcript and protein levels (*p* < 0.001), but the same number of decidual cells (*p* > 0.05). *GR*/*NR3C1* availability was reduced in PUI (*p* < 0.05), along with decreased placental cortisol-to-cortisone conversion. Our findings suggest that PUI could be associated with disturbances of the parturition cascade, possibly due to inadequate P4-PGR and glucocorticoid signaling in the placenta.

## 1. Introduction

Parturition is a complex process requiring precise communication between the fetus and the mother. The biological processes involved in canine parturition have been thoroughly studied in recent years, leading to progress in understanding its underlying mechanisms and further highlighting the distinct reproductive physiology of the dog. The dog is the sole domestic animal in which there is no steroidogenic activity in the placenta, with progesterone (P4) deriving entirely from the corpus luteum (CL) [1,2]. This means that there is no pregnancy- or parturition-associated increase in estradiol (E2) observed in this species [1,3,4]. Following the initially high circulating levels of P4, the peripheral concentrations of luteal steroids begin to decline during the second half of gestation as CL function gradually decreases [5]. Once P4 reaches the low threshold of approximately 2–3 ng/mL, it undergoes a further sharp drop, indicating prepartum luteolysis, 12–24 h before parturition [6,7,8,9]. Although the triggers for luteolysis are not yet fully understood, they seem to be associated with placental feto-maternal communication, related to P4 signaling via its nuclear receptor PGR in response to the decreasing availability of the ligand [6]. Consequently, the prepartum P4 withdrawal leads to the activation of placental prostaglandin synthesis, resulting in increased circulating levels of PGF2α originating from the fetal trophoblast [6,7]. This is accompanied by a strong apoptotic and pro-inflammatory response in the CL that indicates active functional and structural luteolysis (reviewed in [7]). The rapid luteolytic P4 decrease is associated with decreased expression, and presumably activity, of utero-placental HPGD (prostaglandin dehydrogenase) [4]. HPGD likely facilitates the passage of prostaglandins from the placenta to the myometrium and thus enables uterine contractions and the onset of labor [4]. The proposed myometrial source of PGF2α [10,11] was recently confirmed, with an indirect myocontractile activity of PGF2α being suggested [12]. Finally, the local placental withdrawal of P4/PGR signaling results in placentolysis and the expulsion of fetal membranes.

The sensitivity of the placenta to P4 is inseparably associated with decidual cells. Decidual cells are stroma-derived and arise from the morpho-functional mesenchymal-epithelial transition of endometrial fibroblasts, a process referred to as decidualization, which is embryo-induced in dogs [13]. These are the only cells in the canine placenta that express the nuclear P4 receptor (PGR) [6,14], and constitute a key functional component of the feto-maternal communication during the initiation of the parturition cascade. Thus, the natural or antigestagen-mediated decrease in P4-PGR signaling in decidual cells impairs their function both in vivo and in vitro [6,15], leading to the prepartum release of PGF2α by the trophoblast [4,6]. The molecular interaction between decidual cells and the trophoblast that regulates the secretory activity of the trophoblast remains to be elucidated. There is also a concomitant increase in the local placental cortisol-synthesizing capacity, mirrored by the lowered expression and microsomal activity of the cortisol to cortisone convertase HSD11B2 (inactivating cortisol) [16]. This leads to a functional shift in the ratio between HSD11B1 (cortisol activator) and HSD11B2 towards cortisol production [16]. The placental and decidual expression of HSD11B2 is PGR-dependent [15,17]. From a clinical perspective, it is important to note that while elevated circulating cortisol levels have been observed during canine parturition, their significant variability prevents the use of cortisol as a reliable predictor for the initiation of labor [8,18,19,20]. Consequently, the local increase in placental cortisol may not necessarily be reflected in higher circulating levels found in maternal blood [16]. This further highlights the crucial role of local placental mechanisms—particularly the activities of HSD11B1 and HSD11B2—in regulating glucocorticoid activity within the placenta.

Moreover, although prepartum PGF2α release and altered HSD11B2 activity are associated with an increase in the local expression of the glucocorticoid receptor (*GR*/*NR3C1*) in the trophoblast during natural parturition, this is not the case during antigestagen-induced luteolysis and placentolysis, shown in mid-gestation, where the blockage of PGR induces placental PGF2α release without affecting *GR*/*NR3C1* expression [21]. This suggests that elevated GR levels are not required for placental PGF2α synthesis and that *GR*/*NR3C1* expression is independent of PGR. Other functions have been proposed for *GR*/*NR3C1*, particularly during the local withdrawal of P4 activity, as P4 shows affinity for this receptor and, in humans, *GR*/*NR3C1* has been shown to compete with PGR for the ligand [22].

Apart from the removal of the P4 block, the mechanisms by which signaling of the parturition cascade are transmitted from the placenta to the myometrium for timely initiation or support of its contractility remain unclear. Accordingly, parturition may be disrupted by various complications, with dystocia representing a significant clinical issue. This condition is characterized by abnormal or difficult labor and can result from anatomical, physiological, or genetic factors [23,24]. Primary uterine inertia (PUI) prevents the uterus from initiating or sustaining effective labor contractions, despite the presence of an unobstructed birth canal. It is the leading cause of dystocia in dogs, accounting for as many as 59% of cases, highlighting its significance among parturition complications [25]. Despite several factors having already been considered, the pathophysiology of PUI in dogs is still not well understood, as the functional and molecular alterations remain largely undefined. For example, although PUI is associated with little or no myometrial contractility, an increase in the transcriptional availability of the oxytocin receptor (OXTR) was observed in the myometrium of dogs with PUI, compared with obstructive dystocia [26]. Altered uterine PGR expression was also considered as a possible cause of PUI, yet the results were inconclusive [26]. Furthermore, circulating levels of ionized calcium and glucose did not differ between animals with PUI and those with other causes of dystocia [27], nor were there compelling results related to their influence on uterine prostaglandin production [28]. Consequently, due to the frequent failure of pharmaceutical treatments to resolve PUI, even those including oxytocin or calcium [23,29], emergency Cesarean sections are necessary in more than 60% of canine dystocia cases [23,25,29,30]. Therefore, further research and a deeper understanding of the molecular mechanisms associated with PUI are needed for clinical advancement and better treatment strategies. The existing studies of the pathophysiology of PUI focused on comparing it with obstructive dystocia, but did not provide a direct comparison to normal parturition in dogs, which is essential for fully understanding the hormonal and molecular differences that regulate the normal labor process. Further, those studies did not consider the importance and potential involvement of glucocorticoids and their receptors in PUI, highlighting a gap in our understanding of the role of glucocorticoid regulation in abnormal labor processes. Similarly, less focus has been placed on the placenta as the organ where direct contact and the initiation of the parturition cascade take place. 

Therefore, the aim of the present study was to investigate the potential dysregulation of the parturition cascade in dogs with PUI. We compared the placental availability of key factors involved in the parturition process between dogs with PUI and those with natural prepartum luteolysis, in which the prepartum signaling cascade was initiated. Our analyses included the expression of prostaglandin-related enzymes (PGFS/AKR1C3 (PGF2α synthase), PTGS2/COX2 (prostaglandin-endoperoxide synthase 2), PTGES (PGE2 synthase), HPGD (15-prostaglandin dehydrogenase)), the expression of the glucocorticoid receptor *GR*/*NR3C1*, as well as expression of HSD11B1/-2 and placental microsomal activity of HSD11B2. Finally, we assessed the expression of the nuclear PGR. These genes were not selected in a data-driven or exploratory manner but were chosen a priori based on previous literature and their established biological relevance to the mechanisms involved in the canine parturition cascade.

## 2. Materials and Methods

### 2.1. Animals, Tissue Collection and Preservation

The samples utilized in this study were used previously by our research group and by our collaborators [6,16,31,32], in accordance with ethical guidelines for the use of animal samples in research. They were obtained from bitches of different breeds (*n* = 8) that either presented with dystocia and were subsequently diagnosed with primary uterine inertia (PUI, *n* = 4; two Maremmano Sheepdogs, one German Shepherd and one Maltese) or were undergoing natural prepartum luteolysis (LUT, *n* = 4). Animals in the LUT group were mixed-breed, thus, not belonging to a specific breed frequently associated with higher predisposition for PUI. In LUT, routine ovariohysterectomy was performed when P4 levels dropped below 3 ng/mL in three consecutive measurements, with regular measurements (every 6 h) starting on day 58 of pregnancy. The PUI animals failed to deliver any neonates at full term, presenting no or only very weak uterine and abdominal contractions observed on tocodynamometry and upon digital vaginal stimulation (feathering) [31,32]. Further details on the diagnosis of PUI for these animals can be found in previous publications [31,32]. Obstruction of the birth canal was excluded through digital vaginal examination and/or radiographs. These animals were treated with an emergency Cesarean section that was performed solely based on medical necessity. The PUI samples were collected after the surgical delivery of all puppies, followed by the removal of the uterus through ovariohysterectomy. Tissue samples were washed with phosphate-buffered saline (PBS) and dissected to remove connective tissue. For immunohistochemistry (IHC), the paraffin blocks with entire utero-placental compartments were used, with staining being focused on the placenta. The qPCR, Western blot and the isolation of microsomes involved only the placenta, which was macroscopically dissected from the uterus. Therefore, the tissue material was either fixed in 10% neutral phosphate-buffered formalin for 24 h at 4 °C, washed in frequently changed PBS, dehydrated in a graded ethanol series and embedded in paraffin, or incubated in RNAlater (Thermo Fisher Scientific AG, Reinach, Switzerland) for 24 h at 4 °C and subsequently stored at −80 °C until use. Due to limitations on tissue availability, one placenta from each animal was used in the following analyses.

### 2.2. RNA Isolation, Reverse Transcription and Semi-Quantitative Real-Time TaqMan PCR 

Total RNA was isolated from placental samples using TRIzol reagent (Invitrogen, Carlsbad, CA, USA) according to the manufacturer’s protocol. Total concentration and purity of the isolated RNA were assessed using a NanoDrop 2000 spectrophotometer (Thermo Fisher Scientific AG). RNA integrity was assessed with the Agilent 2200 TapeStation System (Agilent Technologies, Santa Clara, CA, USA). All RNA integrity numbers (RIN) ranged between 8.0-9.4. Possible contaminating genomic DNA was removed with the RQ1 RNase-free DNase kit (Promega, Dübendorf, Switzerland) following the manufacturer’s instructions. Reverse transcription was carried out using random hexamers as primers and reagents from the MultiScribe Reverse Transcriptase kit (Applied Biosystems by Thermo Fisher, Foster City, CA, USA). DNase treatment and reverse transcription were performed using an Eppendorf Mastercycler thermocycler (Vaudaux-Eppendorf AG, Basel, Switzerland).

Semi-quantitative real-time (TaqMan) PCR was applied following the previously published protocol [6]. In short, PCR reactions were conducted in duplicate using Fast Start Universal Probe Master (Roche Diagnostics AG, Basel, Switzerland) in an ABI PRISM 7500 Sequence Detection System fluorometer (Applied Biosystems). Commercially available TaqMan systems were purchased from Applied Biosystems. When predesigned systems were not available, primers and probes were self-designed and ordered from Microsynth (Balgach, Switzerland). Probes were labeled with the reporter dye 6-carboxyfluorescein (FAM) at the 5′-end and 6-carboxytetramethyl-rhodamine (TAMRA) at the 3′-end. All TaqMan systems were tested for their specificity and efficiency, and had been used in our previous projects. A full list of primers and probes is presented in Table 1. Relative gene expression was determined using the comparative CT method (ΔΔCT method). The sample with the lowest detectable transcript amount was used as the calibrator. Three reference genes, PTK2, EIF4H, and KDM4A, previously described to be stably expressed in canine reproductive tissues [33], were used for relative quantification of gene expression. Negative control reactions were performed using autoclaved water or non-reverse transcribed RNA.

### 2.3. Immunohistochemistry Staining and Image Analysis 

The standard immunoperoxidase method was employed, using previously published protocols [6,16] with some modifications. Briefly, prepared tissue sections were deparaffinized in xylene, rehydrated in a graded ethanol series and washed in running tap water. For antigen retrieval, slides were heated at 100 °C (270 W) using a microwave oven for 15 min in either 10 mM citrate buffer, pH 6.0 (for GR, PGFS and PGT), or EDTA buffer, pH 9.0 (0.1 M Tris base + 0.01 M EDTA solution; for HSD11B2 and PGR). Endogenous peroxidase activity was quenched using 0.3% hydrogen peroxide in methanol for 30 min at ambient temperature. Nonspecific binding was blocked for 30 min with either 10% normal goat serum or 10% normal horse serum, depending on the secondary antibody used. Slides were then incubated overnight at 4 °C with the primary antibody at the following dilutions: anti-HSD11B2 (custom-made [16]; Eurogentec, Seraing, Belgium) at a 1:2000 dilution; anti-GR (LS66578, LSBio, Shirley, MA, USA) at a 1:50 dilution; anti-PGT (sc103085, Santa Cruz Biotechnology Inc., Santa Cruz, CA, USA) at a 1:50 dilution; anti-PGR (IM1408, Beckman Coulter Life Sciences, Indianapolis, IN, USA) at a 1:75 dilution; and anti-PGFS (custom-made [4]; Eurogentec, Seraing, Belgium) at a 1:750 dilution. There was no species-specific antibody available for the detection of HSD11B1 [16]. Negative/isotype controls were performed by replacing the primary antibody with non-targeting IgGs (isotype control) diluted to the same concentration as the primary antibodies: mouse IgG2a (Exbio Clone 11-794-c100 MOpC-173, Vestec, Czech Republic) for GR and PGR, or goat IgG (I-5000, Vector Laboratories Inc., Burlingame, CA, USA) for PGT. For HSD11B2 and PGFS, pre-immune sera from the animals used to generate the respective antibodies were used [4,16]. The biotinylated secondary antibodies were applied for 30 min (goat anti-guinea pig IgG BA-7000, 1:200; goat anti-mouse IgG AI-9200, 1:500; horse anti-goat IgG BA-9500, 1:200), followed by the application of a streptavidin-peroxidase Vectastain ABC kit (all from Vector Laboratories Inc.). Positive signals were revealed using the Liquid ImmPACT DAB substrate kit (SK-4105, Vector Laboratories Inc.). Slides were then counterstained with hematoxylin, washed under running tap water, dehydrated and mounted with Pertex mounting medium (Histolab, Askim, Sweden). Samples from all animals involved in the study were stained. To avoid possible batch differences, staining was performed simultaneously in all specimens for each of the antibodies used. The localization of positive signals and the capture of representative images were carried out using a Leica DMRXE light microscope equipped with a Leica DFC425 camera (Leica Microsystems, Wetzlar, Germany). 

Pictures from four random fields in the placenta at 400× magnification were used to quantify the optical density of DAB staining obtained from the anti-PGR antibody. For this, captured images were uploaded into FIJI software (ImageJ version 2.9.0, US National Institutes of Health, Bethesda, MD, USA). The separation of the DAB channel (color 2–brown) from the images was obtained with the Colour Deconvolution 1.7 plugin with H DAB vectors. Then, the mean grey value of the DAB channel was measured for each image. Relative optical density of DAB, i.e., the average darkness attributable to DAB staining, was calculated by dividing the mean grey value by the maximum intensity of the respective image. 

The number of decidual cells (PGR-positive) in the placenta was quantified using a Keyence VHX-6000 brightfield microscope (Keyence Deutschland GmbH, Neu-Isenburg, Germany). Cells with positive staining for anti-PGR were identified and counted in five random fields from the center area of the placental labyrinth at 200× magnification. The average number of decidual cells per field was then calculated for each group. 

### 2.4. Protein Extraction and Western Blot 

The relative protein expression of PGR in the placenta was evaluated following our previously published protocol [4]. Placental samples were homogenized using an IKA Euro ST-D overhead stirrer (IKA-Werke GmbH, Staufen, Germany) in a lysis buffer (Net2 Buffer: 50 mM Tris–HCl, pH 7.4, 300 mM NaCl, 0.05% NP-40) supplemented with 10 μL/mL of a protease inhibitor cocktail (Sigma-Aldrich Chemie GmbH, Buchs, Switzerland). The homogenates were then centrifuged at 10,000× *g* for 10 min to sediment tissue debris and the protein contents were determined using the Bradford assay on a SmartSpec Plus spectrophotometer (Bio-Rad Laboratories, Munich, Germany). A sample buffer (25 mM Tris-Cl, pH 6.8, containing 1% SDS, 5% β-mercaptoethanol, 10% glycerol, and 0.01% bromophenol blue) was used to normalize protein concentration. A total of 20 μg of protein from each sample was used for electrophoresis on a 12% polyacrylamide gel (AppliChem GmbH, Darmstadt, Germany). Proteins were then transferred to a methanol-activated polyvinylidene difluoride (PVDF) membrane (Bio-Rad Laboratories). To block non-specific binding, membranes were incubated with 5% low-fat powdered milk in PBST (PBS with 0.25% Tween-20), and then incubated overnight at 4 °C with anti-PGR antibody, the same as for IHC, at a 1:300 dilution in 2.5% low-fat powdered milk in PBST. Subsequently, the membranes were incubated with a goat anti-mouse horseradish peroxidase (HRP)-conjugated secondary antibody diluted 1:15000 (W402B, Promega). Signals were detected with the Super Signal West Chemiluminescent Kit substrate (Thermo Fisher Scientific AG) in a Chemi-Doc XRS+ System with Image Lab Software (v6.0.1, Bio-Rad Laboratories). For loading control and relative quantification, membranes were submerged in glycine for 1 h and reblotted against BACTIN (sc-69879; dilution 1:1000; Santa Cruz Biotechnology). Band optical density was quantified using ImageJ software (version 1.54m). The relative protein expression was calculated by normalizing the optical density of PGR to that of ACTB on the re-probed membranes, and results are presented as standardized optical density (SOD). Full immunoblots used for the quantification of SOD are presented as Supplemental Figure S1 (Figure S1). Dog uterine stromal (DUS) cells, in which the PGR expression had previously been confirmed, were used as a positive control [13] the DUS proteins were added for the representative Western blot.

### 2.5. Placental Microsomal Cortisol-to-Cortisone Conversion Activity

Having observed decreased mRNA and protein expression of HSD11B2 in PUI, we decided to assess the ability of placental tissue to convert cortisol into its biologically inactive form, cortisone (i.e., HSD11B2 activity). Therefore, microsomal fractions (crude endoplasmic reticulum) were isolated from dissected placental labyrinth using the Endoplasmic Reticulum Isolation Kit (Sigma Aldrich Chemie GmbH), following the manufacturer’s instructions and previously published methodologies [16]. The cortisol to cortisone conversion assay followed the previously published protocol [16]. Reaction mixtures (100 μL volume) were created by combining 50 μL of microsomal preparations, 0.25 mM NAD (Roche Diagnostics, Mannheim, Germany) used as co-factor and 14.2 nM tritium-labeled cortisol as a substrate ([1,2,6,7-3H(N)]-cortisol, PerkinElmer LAS GmbH, Rodgau, Germany). For blank controls, substrate and cofactor were incubated in the absence of microsomal protein. As reference samples with high and low enzyme activity, microsomal proteins were prepared either from uteroplacental tissue collected at the postimplantation stage (day 18–25 of pregnancy) or from myometrium collected at prepartum luteolysis [16]. Immediately after incubation for 20 min at 37 °C, the samples were analyzed as previously described in detail [16]. Briefly, samples were extracted with ethyl acetate, dried in a MicroDancer infrared vortex-evaporator (Hettich AG, Baech, Switzerland), and redissolved in 100 μL of HPLC mobile phase (methanol/acetonitrile/water 43:3:54 *v*/*v*/*v*). Aliquots of 20 µL were then separated via HPLC on a 150 × 4 mm Eurospher II 100–5 C18 reversed-phase column (Knauer GmbH, Berlin, Germany) at a flow rate of 1 mL/min. Eluted 0.5 mL fractions were then collected and evaporated. The 3H-activity in HPLC fractions was then measured after adding scintillation cocktail (Rotiszint ecoplus, Carl Roth GmbH, Karlsruhe, Germany) in a Tri-CARB 4810TR Liquid Scintillation Counter (PerkinElmer LAS GmbH, Rodgau, Germany). Identification of substrate and metabolite was based on a comparison of retention times with authentic tritiated standards. The percentage of substrate conversion was calculated from the distribution of 3H activity among the peaks after subtracting technical background of β-scintillation counter and baseline correction. Besides all biological replicates, four technical replicates were performed for each sample.

### 2.6. Statistical Analysis

Given the small sample size, a formal power analysis was not feasible. The comparison between LUT and PUI groups was assessed with Student’s *t*-test following a normality test; GraphPad 3.06 (GraphPad Software Inc., San Diego, CA, USA) was used. A statistical significance was considered when *p* < 0.05. Numerical results are presented as geometric mean ± geometric standard deviation (TaqMan PCR), or as mean ± standard deviation. False discovery rate (FDR; Benjamini–Hochberg) correction was applied to reduce the risk of Type I errors (results are presented for all genes showing statistically significant differences).

## 3. Results

### 3.1. Placental Transcriptional Availability of Parturition Cascade-Associated Factors

**Figure 1:**
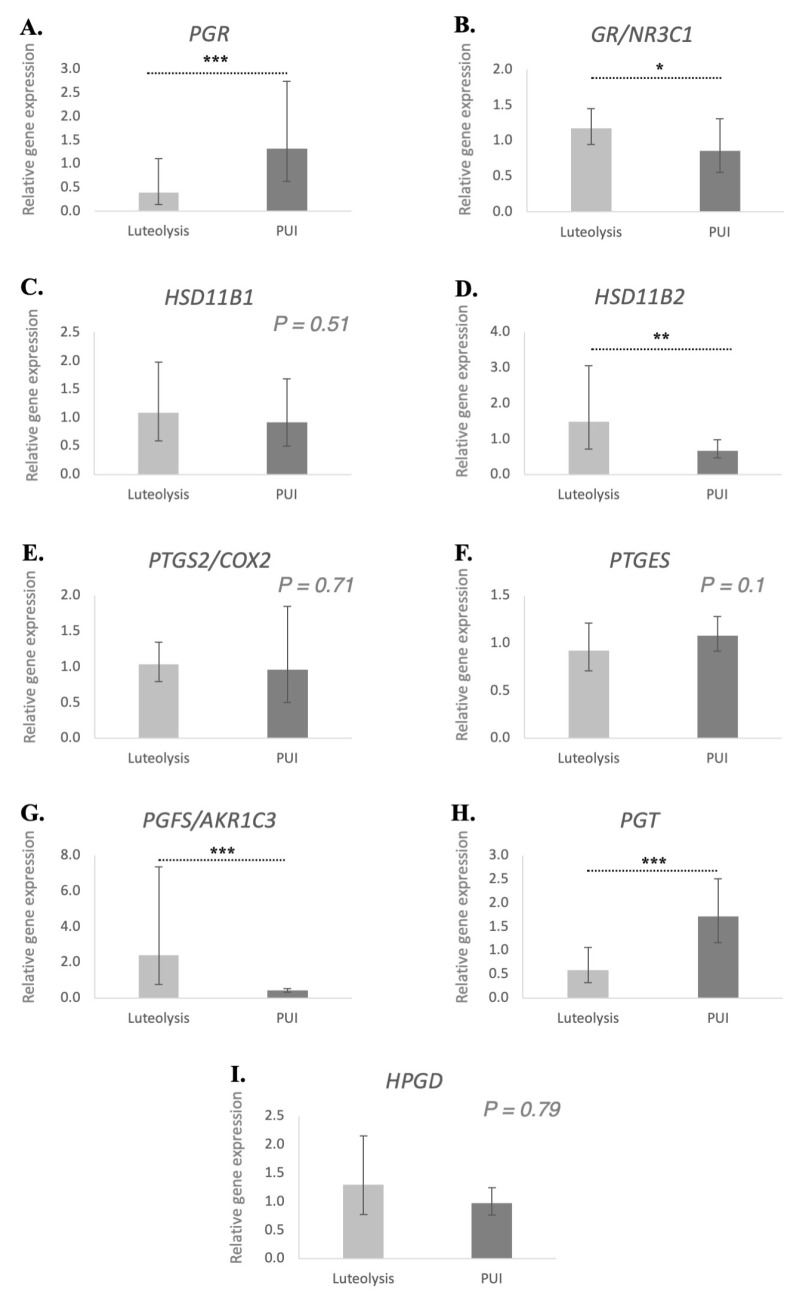
Relative gene expression of *PGR* (**A**), *GR*/*NR3C1* (**B**), *HSD11B1* (**C**), *HSD11B2* (**D**), *PTGS2*/*COX2* (**E**), *PTGES* (**F**), *PGFS*/*AKR1C3* (**G**), *PGT* (**H**) and *HPGD* (**I**) in the placenta of bitches at prepartum luteolysis and presenting with primary uterine inertia (PUI). The relative gene expression was determined using semi-quantitative real-time TaqMan PCR, and the results are expressed as geometric means ± geometric standard deviation. Student’s *t* test was used to compare the detected mRNA availability between luteolysis and PUI groups. Bars with asterisks differ at: * *p* < 0.05, ** *p* < 0.01, *** *p* < 0.001.

The expression of all assessed genes, i.e., *PGR*, *GR*/*NR3C1*, *HSD11B1* and *-2*, *PTGS2*/*COX2*, *PTGES*, *PGFS*, *PGT* and *HPGD*, was detectable in all placental samples investigated (Figure 1). The mRNA availability of *PGR* was significantly higher in the PUI group compared to the normal prepartum luteolysis group (*p* < 0.001; FDR = 0.01). Both *NR3C1* (encoding for the glucocorticoid receptor (GR), *p* < 0.05; FDR = 0.049) and *HSD11B2* (*p* < 0.01; FDR = 0.007) were significantly lower in the placentae of dogs presenting with PUI than in those with normal prepartum luteolysis. Similarly, PGF2α synthase *PGFS*/*AKR1C3* was significantly lower in the PUI group than in prepartum luteolysis samples (*p* < 0.001; FDR = 0.0009). Conversely, the abundance of *PGT* mRNA was significantly higher in animals presented with PUI (*p* < 0.001; FDR = 0.0009). In contrast, no differences (*p* > 0.05) were observed for the transcripts of *HSD11B1* (*p* = 0.51), the prostaglandin synthases, *PTGS2* (*p* = 0.71) and *PTGES* (*p* = 0.1), along with dehydrogenase *HPGD* (*p* = 0.79), which were all similar between the two groups.

### 3.2. Immunohistochemistry 

**Figure 2:**
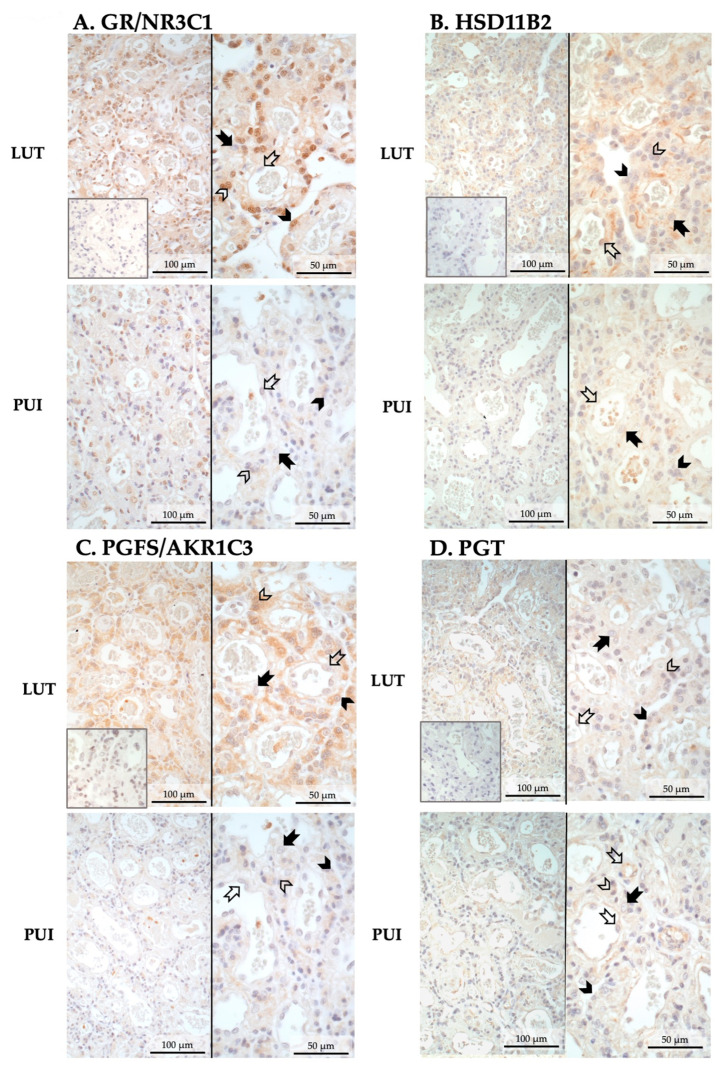
Representative photomicrographs of the immunohistochemical localization of *GR*/*NR3C1*, HSD11B2, PGFS, and PGT in the placental labyrinth of bitches at prepartum luteolysis (LUT) and presenting with primary uterine inertia (PUI). (**A**) GR (encoded by *NR3C1*) and (**C**) PGFS were mainly localized in cyto- and syncytiotrophoblast. (**B**) HSD11B2-positive signals were identified in syncytiotrophoblast and endothelial cells. (**D**) PGT appeared to be mainly localized in placental endothelial cells, with lower signal intensity in the trophoblast, mainly syncytiotrophoblast. No staining was observed in the isotype controls (insets in pictures presented at the same magnification). Solid arrow = decidual cell; open arrow = endothelial cell; solid arrowhead = cytotrophoblast; open arrowhead = syncytiotrophoblast.

IHC was used to assess the expression of those factors associated with the parturition cascade that had altered mRNA expression between both groups, namely *GR*/*NR3C1*, HSD11B2, PGFS/AKR1C3 and PGT. The cellular localization of these factors was the same for the luteolysis and PUI groups. Positive signals for GR (encoded by *NR3C1*) and PGFS were mainly localized in fetal trophoblast cells, both cyto- and syncytiotrophoblast, with GR prevailing in the cytotrophoblast and PGFS/AKR1C3 in the syncytiotrophoblast (Figure 2A,C). Similarly, the anti-HSD11B2 staining appeared to be mainly localized in syncytiotrophoblast cells, with some signals being detectable in endothelial cells (Figure 2B). The intensity of IHC staining for these three factors appeared to follow the expression patterns observed at the mRNA level, i.e., being stronger in prepartum luteolysis samples than in PUI samples (Figure 2A–C). The PGT-positive signals were strongly represented in endothelial cells, followed by positive staining in syncytiotrophoblast cells (Figure 2D). In apparent agreement with the transcriptional findings, the signals in PUI samples, mainly those localized in endothelial cells, seemed stronger than those in LUT (Figure 2D).

### 3.3. PGR Protein Availability in the Placenta at Prepartum Luteolysis and PUI

**Figure 3:**
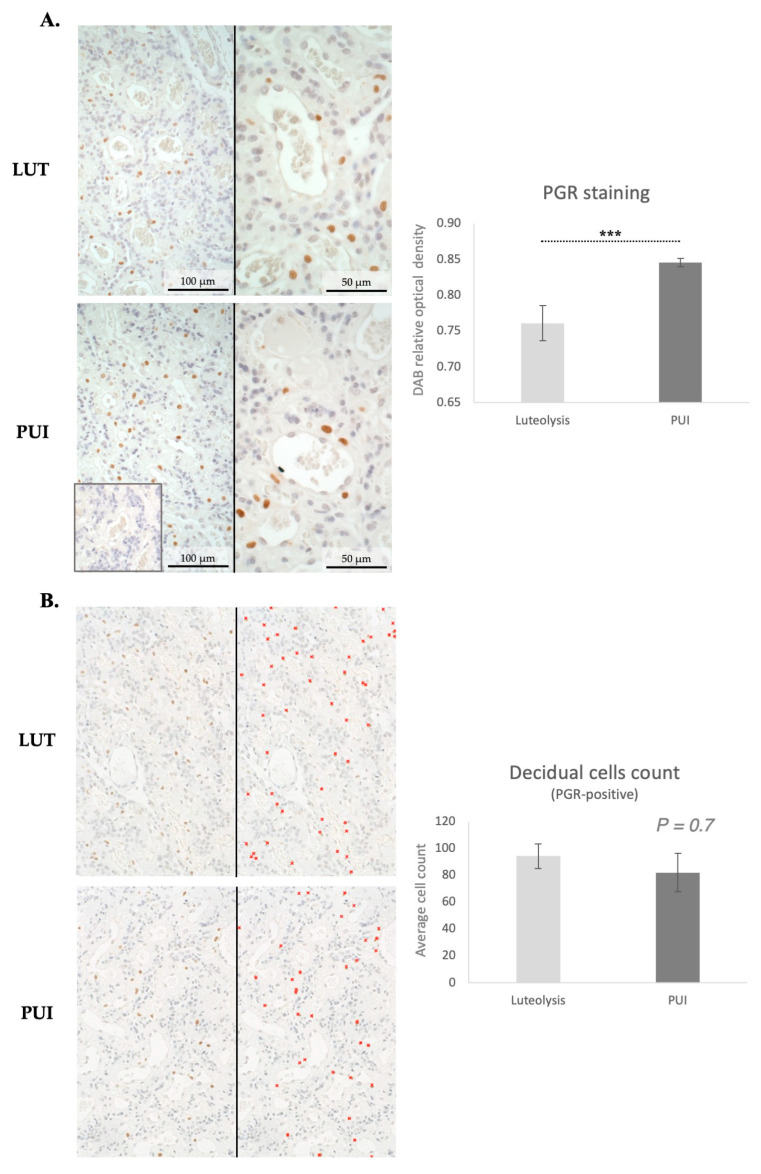
Immunohistochemical staining of PGR in the placental labyrinth of bitches at prepartum luteolysis (LUT) and presenting with primary uterine inertia (PUI). (**A**) Positive signals for the nuclear localization of PGR were observed in decidual cells. No staining was detected in the isotype control (inset in (**A**), at the same magnification). The graph shows the results of the quantification (relative optical density) of DAB-positive staining in four random fields of the placenta (central part of the labyrinth) of each animal. Statistical comparison between LUT and PUI groups was performed with Student’s *t*-test, revealing a *** *p* < 0.001. (**B**) Quantification of PGR-positive decidual cells was performed in five random fields in the central area of placentae from all animals. Photomicrographs were obtained at 200× magnification. Representative images display the IHC staining before (left images) and after quantification (right images), with red crosses marking counted cells. As shown in the graph, no significant difference in the number of decidual cells was observed when comparing LUT and PUI groups, with Student’s *t*-test revealing *p* = 0.7. All numerical values are presented as mean ± standard deviation.

**Figure 4:**
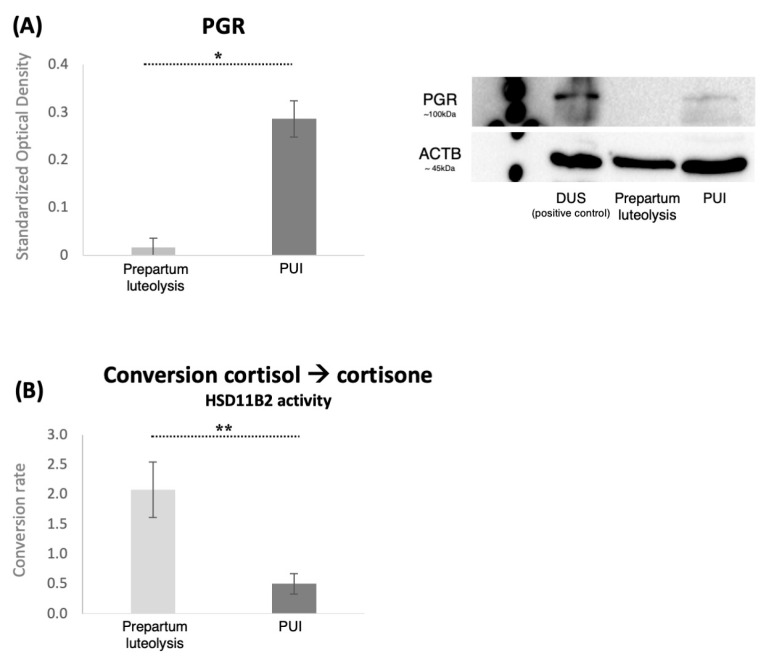
PGR expression and placental conversion rates of cortisol-to-cortisone (HSD11B2 activity). (**A**) Protein expression of PGR in placental homogenates from bitches at prepartum luteolysis or presenting with primary uterine inertia (PUI) as determined by Western blot. Representative immunoblots show the presence of a specific band for PGR (~100 kDa). Membranes were reblotted with anti-ACTB (~45 kDa) antibody that served as a loading control. Proteins isolated from decidualized DUS cells, which have increased availability of PGR [13], were added to the blot to confirm the identity of the PGR-specific band. Standardized optical density, represented as mean ± standard deviation, was measured in all samples. The statistical difference between the two groups was assessed with Student’s *t*-test, revealing * *p* < 0.0001. Full immunoblots are presented as Supplemental Figure S1 (Figure S1). (**B**) The assessment of HSD11B2 activity in placental microsomes, comparing the cortisol-to-cortisone conversion capacity between the primary uterine inertia (PUI) and LUT groups. The data were obtained by subtracting the background signal to ensure accurate measurements of enzymatic activity, with all values exceeding the background level being considered. Biological and technical replicates are included to demonstrate the reliability of the results. An unpaired, two-tailed *t*-test was performed, yielding ** *p* = 0.03 between the groups. All numerical values are presented as mean ± standard deviation.

Considering the importance of decidual cells in the onset of the parturition cascade, expression of PGR in the placental labyrinth was assessed both by immunohistochemistry and by Western blot. PGR was detected in decidual cells both during prepartum luteolysis and in dogs presenting with PUI (Figure 3A). The decidual cell-specific PGR-positive signals appeared stronger in PUI than in luteolysis, apparently following the mRNA expression patterns. The physiological regulatory importance of PGR for parturition prompted us to determine the relative optical density of DAB staining, which confirmed higher PGR protein levels in PUI than in dogs at prepartum luteolysis (*p* < 0.001, Figure 3A). To assess whether the increased availability of PGR at PUI was associated with an increased number of decidual cells, PGR-positive cells were quantified in both groups. A total of five fields from the central area of the placental labyrinth were quantified for each animal. However, no significant differences in the number of decidual cells were observed between prepartum luteolysis and PUI (*p* = 0.7, Figure 3B). Next, to support the densitometry findings of the IHC staining, quantification of placental PGR protein expression was performed utilizing Western blot, which revealed significantly higher PGR levels in PUI, matching the mRNA and IHC findings (*p* < 0.001, Figure 4A). 

### 3.4. Placental Microsomal Cortisol-to-Cortisone Conversion Capacity 

The capacity of the placental microsomes to inactivate cortisol locally, i.e., convert cortisol to cortisone via microsomal HSD11B2 activity, was compared between the PUI and the normal prepartum luteolysis groups. The evaluation included all values exceeding the background readouts, as well as both biological and technical replicates. Approximately 90% (±1.7% in four technical replicates) of the substrate was converted into cortisone in the control sample derived from early pregnancy, whereas the luteolysis-derived myometrium showed a significantly lower conversion rate of 1.6% (±0.5% between the technical replicates). These results were expected based on a previous report [16], and served as controls for validating the assay. Although generally low, the placental conversion of cortisol to cortisone was significantly higher during normal prepartum luteolysis compared to PUI (*p* = 0.03, Figure 4B). The PUI values were lower than those determined for the myometrium.

## 4. Discussion

The pathophysiology of PUI, a major cause of dystocia in dogs, remains unresolved. Most of the current literature has focused on the local changes within the uterus, with less attention given to the placenta, even though the placenta is the part of the feto-maternal interface where the parturition cascade is initiated in response to fetal maturation. Further, those studies primarily compared PUI with obstructive dystocia. In response to these knowledge gaps, we determined the placental profiles of factors known to be involved in the parturition cascade, comparing clinical samples of PUI and natural prepartum luteolysis. The particular focus was on the nuclear P4 receptor (PGR), cortisol metabolism and the glucocorticoid receptor *GR*/*NR3C1*, as well as on some of the factors related to the prostaglandin system. Importantly the presented findings were interpreted not solely on the basis of *p*-values, but also by considering the functional relevance of the genes involved.

During natural prepartum luteolysis, the supply of placental PGF2α appears to be regulated by an increase in PTGS2/COX2 availability, with downstream synthesis involving the conversion of PGE2 to PGF2α, dependent on enhanced PTGES activity [6]. In contrast, the expression of PGFS (AKR1C3) decreases prepartum compared to earlier stages of pregnancy [4,6]. At the local level, prostaglandin activity is accentuated by the upregulation of the PGF2α receptor (PTGFR) and its corresponding transporter, PGT [6,34]. The basal supply of prostaglandins in the placenta of PUI dogs did not appear to differ from normal prepartum luteolysis, as indicated by the stable *COX2* and *PTGES* expression observed here. This is also in agreement with previous findings focusing on the uterine availability of prostaglandins comparing PUI to obstructive inertia [28]. Nevertheless, the reduced expression of *PGFS* in the placenta of dogs affected by PUI may suggest disturbances in local, i.e., placental, prostaglandin production. Importantly, the lower availability of PGFS was also apparent at the protein level and was accompanied by an increase in *PGT* expression. The elevated expression of *PGT* was interpreted as a possible compensatory mechanism that could be associated with the reduced availability of prostaglandins or altered local signaling mediated by other related factors. Further investigations are required to elucidate these mechanisms. The process by which PGF2α is transferred from the placenta to the uterus remains to be investigated, however, the involvement of PGT cannot be ruled out. Its expression is widely distributed throughout the utero-placental compartment, being localized in endometrial superficial and deep uterine glands, as well as in vascular endothelial cells [34]. The localization of PGT in the fetal trophoblast and endothelial compartments of both the *placenta fetalis* and *placenta materna* was re-emphasized in the present study. 

Next, the HPGD is responsible for the deactivation of prostaglandins PGF2α and PGE2 to their inactive metabolites PGFM and PGEM, respectively [4], and was proposed to act as a gatekeeper of the supply of prostaglandins in the canine pregnant uterus [4]. However, we did not observe any difference in its basal availability between PUI and normal prepartum luteolysis placenta samples. The extent to which HPGD contributes to the passage of PGF2α from the placenta to the uterus needs to be further studied, but based on the present results, it does not seem to be involved in the development of PUI. 

It must be noted that the timing of pregnancy/delivery does not fully correspond between both groups. Animals from the LUT group were in the beginning stages of the parturition cascade, marked by the onset of luteolysis, whereas PUI animals were assumed to be at the expulsion phase. This could, at least in part, be responsible for the transcriptional differences seen in PG-associated factors. 

The P4 withdrawal in the canine placenta coupled with the reduction in PGR activity represents a critical signaling event in the initiation of parturition in dogs [6,7]. In humans, the balance of two nuclear P4 receptor isoforms, PRA and PRB (approx. 90 kDa and 120 kDa, respectively; reviewed in [35]), is critical for the initiation of labor. Thus, PRB predominates during pregnancy to maintain uterine quiescence, but as parturition approaches, increased PRA expression facilitates functional P4 withdrawal by repressing PRB activity and reducing P4 signaling [35,36,37,38]. This process is further enhanced by inflammatory stimuli, which stabilize PRA and amplify its transrepressive effects, promoting the transition to labor [36,39]. The placental presence of the two distinct PGR isoforms has not yet been confirmed in the dog, nor in dog uterine stromal (DUS) cells, which are utilized as a model in our laboratory for investigating the canine-specific decidualization process [13,40]. The antibody used in our laboratory targets a recombinant protein corresponding to amino acids 922–933 of human PGR, recognizing both major PGR isoforms in humans. There is a distinct band, corresponding to a protein of approximately 100 kDa, seen in immunoblots performed with proteins isolated from DUS cells [40], which were also used as a positive control in this study, as well as in immunoblots of placental tissue. The specificity of this band has been confirmed in studies using antigestagens [40]. Our, as-yet-unpublished, results suggest that the progressive decline in decidual cell numbers during pregnancy may contribute to reduced P4/PGR signaling, potentially as a part of placental maturation toward parturition. Alongside the decreasing luteal P4 availability, this indicates an additional factor influencing P4/PGR signaling beyond the pregnancy-associated decline in P4 concentration.

We found a higher transcriptional availability of PGR in placentae from PUI than in those undergoing normal prepartum luteolysis. This was further reflected at the protein level by increased immunostaining intensity of PGR and higher PGR protein expression, determined by Western blot. Interestingly, however, no differences were observed in the number of PGR-expressing decidual cells, indicating that decreased PGR expression is not related to the delayed withdrawal of decidual cells. The increased PGR expression in the PUI placentae could suggest enhanced PGR/P4 activity, which also implies that the functional withdrawal of P4 in the placenta, mediated by PGR expression in decidual cells, could be impaired or delayed in this group. The prepartum P4/PGR withdrawal induces immune system responses in the placenta, under both normal and antigestagen-induced parturition/abortion [17]. These responses also involve decidual cells, as implied by the studies using decidualized DUS cells [15]. The extent to which the physiological changes within the immune system-associated regulatory mechanisms of the placenta that occur during the natural withdrawal of PGR activity could also apply to disturbances leading to PUI or other cases of dystocia remains to be elucidated.

Concomitant with the aberrantly increased placental PGR expression in PUI, we found decreased *GR*/*NR3C1* expression. Together, this suggests a functional and regulatory dysregulation in both the maternal and fetal placental cellular compartments: while the *placenta materna* fails to downregulate PGR expression, the *placenta fetalis* does not increase GR expression. The interaction between PGR and GR is not clear, nor can causality be confirmed at this time, especially since blocking PGR activity with antigestagens does not affect GR expression [21]. It does, however, initiate placental prostaglandin production, which depends upon PGR withdrawal [6,41]. Although increased *GR*/*NR3C1* availability is not required for the prepartum release of PGF2α in dogs, the term-specific upregulation of GR indicates that glucocorticoids might play a paracrine role in facilitating placental P4 withdrawal [21], possibly through interference with the PGR via interaction with its natural ligand [22]. Additionally, glucocorticoids might modulate the placental immune response, potentially indirectly, influencing P4 signaling [42].

An interesting finding from this study was the decreased microsomal conversion activity toward inactivating cortisol in PUI compared with normal prepartum luteolysis, indicating reduced HSD11B2 activity, possibly increasing local cortisol levels in PUI. This was in accordance with the decreased transcriptional and translational availability of HSD11B2 in fetal trophoblasts, and was interpreted as indicative of a compensatory mechanism in response to lowered local, i.e., placental, cortisol signaling, possibly due to the decreased GR expression. The localization of GR within the trophoblast underscores the importance of fetal-derived signals in orchestrating the cascade that triggers parturition. A reduced or dysregulated signal from the fetus may weaken the stimulus for myometrial activation, potentially delaying or attenuating the onset of labor. Further research is needed to explore the functional implications of these findings, particularly with regard to the placental role of glucocorticoids in the parturition cascade in dogs. Such studies could provide deeper insights into how glucocorticoid metabolism, receptor activity, and P4 withdrawal regulate the timing and progression of parturition. Also, the more frequent occurrence of PUI in animals with a low number of fetuses [29,43,44] further supports this idea, as an adequate trigger for parturition might not be generated due to insufficient stimulus, which is likely linked to the reduced presence or activity of total fetal signals. The number of fetuses, as well as comparisons between different placentas from the same individual, could not be considered in the present study due to limitations in material availability, but these remain important issues to be addressed in future research. Similarly, the effect of fetal sex could not be considered, as there is no information available in dogs regarding its potential impact on the parturition cascade and placental functionality. Furthermore, the use of a larger number of animals, representing a stronger power analysis, should also be implemented in possible future studies. Moreover, the maturation of trophoblasts, the signaling cascade, and the crosstalk with maternal decidual cells could be disrupted due to insufficient signaling when only a single fetus is present. This lack of adequate communication could impair the functional maturation of trophoblasts, ultimately hindering the signaling pathway necessary for the correct progression of labor and its timely initiation.

## 5. Conclusions

**Figure 5:**
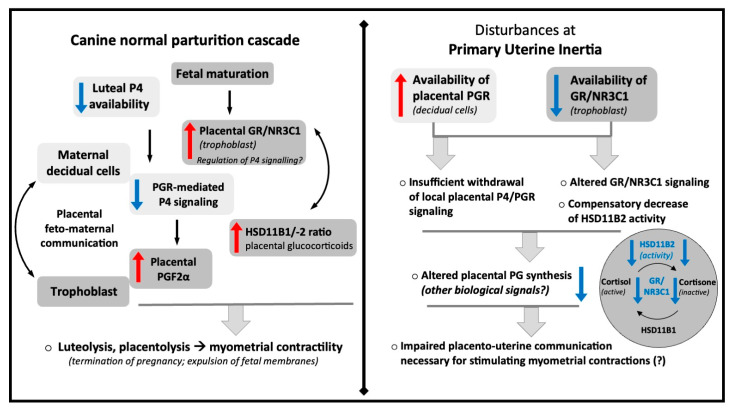
Schematic representation of the most important findings of the current study and the hypothetical mechanisms contributing to the development of primary uterine inertia.

The main findings from this study, including the main physiological deviations observed with PUI and the hypothetical mechanisms possibly contributing to its development, are summarized in the schematic Figure 5. In brief, the study reveals two key divergences from the physiological parturition cascade in PUI. Firstly, PGR expression in the placenta is not sufficiently decreased, but it does maintain normal morphological maturation towards prepartum luteolysis, as both types of placentae have similar morphology and numbers of PGR-expressing decidual cells. Secondly, fetal expression of *GR*/*NR3C1* is not sufficiently increased, which is associated with locally decreased deactivation of cortisol as a compensatory mechanism. Cumulatively, these findings support the hypothesis that the parturition cascade is altered in PUI, which could contribute to the development of this pathology: the lack of a prepartum placental PGR decrease could lead to imbalances in prostaglandin production, associated with altered GR expression and its signaling. In turn, altered prostaglandin production may subsequently impair myometrial activity. However, these causalities remain to be verified. While conducted on a small animal sample size, our analyses yielded statistically meaningful findings for selected factors, providing important insights into potential aberrations contributing to PUI. In this context, our findings underscore the possible role of dysfunctional placental signaling in disrupting the normal processes that initiate parturition. Despite limitations related to possible discrepancies in stage or timing of parturition, this study opens new avenues for research into the pathophysiology of PUI. 

Understanding how disturbances in P4-PGR signaling, prostaglandin synthesis, and glucocorticoid activity influence the initiation of labor could lead to better management strategies for PUI in dogs, which is of great clinical importance. Future investigations into these hormonal pathways and their interactions will be essential to elucidate their roles in pregnancy and parturition, and, ultimately, to improve clinical outcomes for animals affected by PUI.

## Figures and Tables

**Table 1:** List of primers and TaqMan probes used for PCR.

Gene	Name	Accession Numbers	Primer and Probe Sequence for Semi-Quantitative Real Time PCR		Product Length (bp)
*HPGD* (*PGDH*)	Hydroxyprostaglandin dehydrogenase	NM_001284477	Forward	5′-GGC AGC GAA TCT CAT GAA CAG-3′	93
			Reverse	5′-TCT TCT TTC TCA ATG GAT TCA AGGA-3′	
			TaqMan probe	5′-TGA ATG CCA TTT GCC CAG GCT TTG T-3′	
*PGT*	Prostaglandin transporter	NM_001011558	Forward	5′-TGC AGC ACT AGG AAT GCT GTT C-3′	116
			Reverse	5′-GGG CGC AGA GAA TCA TGG A-3′	
			TaqMan probe	5′-TCT GCA AAC CAT TCC CCG CGT G-3′	
*PTGS2* (*COX2*)	Prostaglandin-endoperoxide synthase 2 (=Cyclooxygenase 2)	HQ110882	Forward	5′-GGA GCA TAA CAG AGT GTG TGA TGT G-3′	87
			Reverse	5′-AAG TAT TAG CCT GCT CGT CTG GAA T-3′	
			TaqMan probe	5′-CGC TCA TCA TCC CAT TCT GGG TGC-3′	
*PTGES*	Prostaglandin E synthase	NM_001122854	Forward	5′-GTC CTG GCG CTG GTG AGT-3′	89
			Reverse	5′-ATG ACA GCC ACC ACG TAC ATC T-3′	
			TaqMan probe	5′-TCC CAG CCT TCC TGC TCT GCA GC-3′	
*AKR1C3* (*PGFS*)	Prostaglandin F synthase	NM_001012344	Forward	5′-AGG GCT TGC CAA GTC TAT TGG-3′	74
			Reverse	5′-GCC TTG GCT TGC TCA GGA T-3′	
			TaqMan probe	5′-TCC AAC TTT AAC CGC AGG CAG CTG G-3′	
*PGR*	Progesterone receptor	NM_001003074	Forward	5′-CGA GTC ATT ACC TCA GAA GAT TTG TTT-3′	113
			Reverse	5′-CTT CCA TTG CCC TTT TAA AGA AGA-3′	
			TaqMan probe	5′-AAG CAT CAG GCT GTC ATT ATG GTG TCC TAA CTT-3′	
Commercially available Taq Man Systems (Applied Biosystems)					
*HSD11B1*	Hydroxysteroid 11β dehydrogenase 1		Prod. No. Cf02626817_m1		67
*HSD11B2*	Hydroxysteroid 11β dehydrogenase 2		Prod. No. Cf02690463_s1		82
*NR3C1* (*GR*)	Glucocorticoid receptor/nuclear receptor subfamily 3 group C member 1		Prod. No. Cf02627498_m1		118
*PTK2*	Protein tyrosine kinase 2		Prod. No. Cf02684608_m1		104
*EIF4H*	Eukaryotic translation initiation factor 4H		Prod. No. Cf02713640_m1		136
*KDM4A*	Lysine (K)-specific demethylase 4A		Prod. No. Cf02708629_m1		96

## Data Availability

The data presented in this manuscript is available upon reasonable request to the corresponding author.

## Supplementary Materials

The following supporting information can be downloaded at: https://www.mdpi.com/article/10.3390/ani15203043/s1, Figure S1: Full immunoblots used for relative quantification of PGR in the placenta of bitches at prepartum luteolysis and those with primary uterine inertia. Arrows indicate the bands corresponding to PGR (~100 kDa) and ACTB (~45 kDa).
