# Supplementary figures and images for: Primary Uterine Inertia (PUI) in Dogs Is Associated with Impaired Placental Availability of Factors Involved in the Parturition Cascade
Source: Animals (Basel). 2025 Oct 20;15(20):3043. doi: 10.3390/ani15203043 (PMC12561314; doi:10.3390/ani15203043)

Supplemental Figure S1

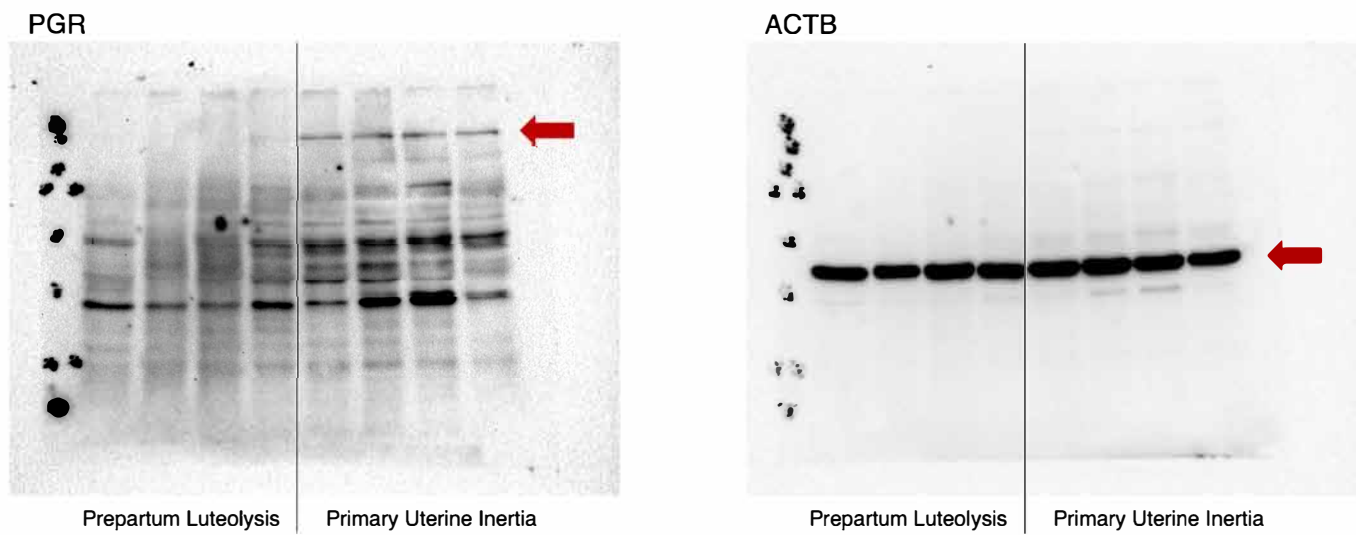

Supplement: Supplementary file 1 [file animals-15-03043-s001.zip › animals-3833399-supplementary.pdf]
